# Coexistence of Graves’ disease and unilateral functioning Struma ovarii: a case report

**DOI:** 10.1186/s12902-015-0060-z

**Published:** 2015-11-04

**Authors:** Tullaya Sitasuwan, Suchanan Hanamornroongruang, Thavatchai Peerapatdit, Nuntakorn Thongtang

**Affiliations:** Division of Endocrinology and Metabolism, Faculty of Medicine Siriraj Hospital, Mahidol University, Bangkok, 10700 Thailand; Department of Pathology, Faculty of Medicine Siriraj Hospital, Mahidol University, Bangkok, 10700 Thailand

**Keywords:** Graves’ disease, Functioning struma ovarii

## Abstract

**Background:**

Coexisting of Graves’ disease and functioning struma ovarii is a rare condition. Although the histology of struma ovarii predominantly composed of thyrocytes, the majority of the patients did not have thyrotoxicosis. The mechanism underlying the functioning status of the tumor is still unclear but the presence of thyroid stimulating hormone receptor (TSHR) is thought to play a role. Here we describe the patient presentation and report the TSHR expression of the tumor.

**Case presentation:**

A 56-year old Asian woman presented with long standing thyrotoxicosis for 23 years. She was diagnosed with Graves’ disease and thyroid nodules. She had bilateral exophthalmos and had high titer of plasma TSHR antibody. Total thyroidectomy was performed and the histologic findings confirmed the clinical diagnosis. The patient had persistent thyrotoxicosis postoperatively. Thyroid uptake demonstrated the adequacy of the thyroid surgery and the whole body scan confirmed the presence of functioning thyroid tissue at pelvic area. The surgery was scheduled and the patient had hypothyroidism after the surgery. The pathological diagnosis was struma ovarii at right ovary. We performed TSHR staining in both the patient’s struma ovarii and in 3 cases of non-functioning struma ovarii. The staining results were all positive and the intensity of the TSHR staining of functioning struma ovarii was the same as that in other cases of non-functioning tumors, suggesting that the determinant of functioning struma ovarii might be the presence of TSHR stimuli rather than the intensity of the TSHR in the ovarian tissue.

**Conclusion:**

In patients with Graves’ disease with persistent or recurrent thyrotoxicosis after adequate ablative treatment, the possibility of ectopic thyroid hormone production should be considered. TSHR expression is found in patients with functioning and non-functioning struma ovarii and cannot solely be used to determine the functioning status of the tumor.

## Background

Coexisting of Graves’ disease and functioning struma ovarii is a rare condition. Struma ovarii is a rare ovarian tumor. Most affected patients are asymptomatic; however thyrotoxicosis from struma ovarii has been reported in 5 % to 15 % of the confirmed cases [1, 2]. Although the histology of struma ovarii predominantly composed of thyrocytes, the majority of the patients do not have thyrotoxicosis. The mechanism underlying the functioning status of the tumor is still unclear. The expression of thyroid-stimulating hormone receptor (TSHR) is thought to play a role [3, 4]. The diagnosis of functioning struma ovarii is challenging especially when the patient had functioning thyroid gland. Here we report an usual case of coexisting Graves’ disease with functioning struma ovarii and the TSHR staining result, including the TSHR staining of the patient with non-functioning struma ovarii.

## Case presentation

A 56-year-old woman presented with persistent thyrotoxicosis. She was first diagnosed with thyrotoxicosis 23 years previously and had been periodically treated with antithyroid drugs for several years at a time. On examination, she had bilateral exophthalmos. Her thyroid gland was enlarged with palpable thyroid nodules. Her serum TSHR antibody level was elevated at 3.86 IU/L (reference range, <1.00 IU/L), thus confirming the diagnosis of Graves’ disease with thyroid nodules.

**Fig. 1:**
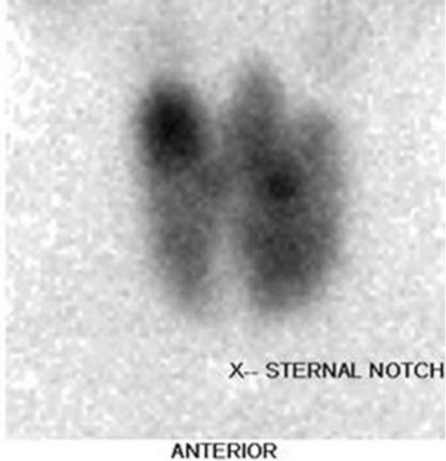
Tc^99^m thyroid scan showed diffusely increased uptake by the thyroid tissue with thyroid nodules

A thyroid scan with Tc^99^m showed generalized increased uptake in the thyroid gland with visualized activity in the pyramidal lobe. One hyperfunctioning nodule at the upper pole of the right lobe and another hypofunctioning nodule in the middle aspect of the right lobe were demonstrated (Fig. 1). The provisional diagnosis at that time was Graves’ disease with thyroid nodules, and ablative treatment was planned. Ultrasound-guided fine-needle aspiration yielded an area of undetermined significance. Total thyroidectomy was performed without perioperative complications. The surgical specimen contained 57.7 g of thyroid tissue. The histological findings supported the clinical diagnosis of Graves’ disease and benign thyroid nodules.

**Fig. 2:**
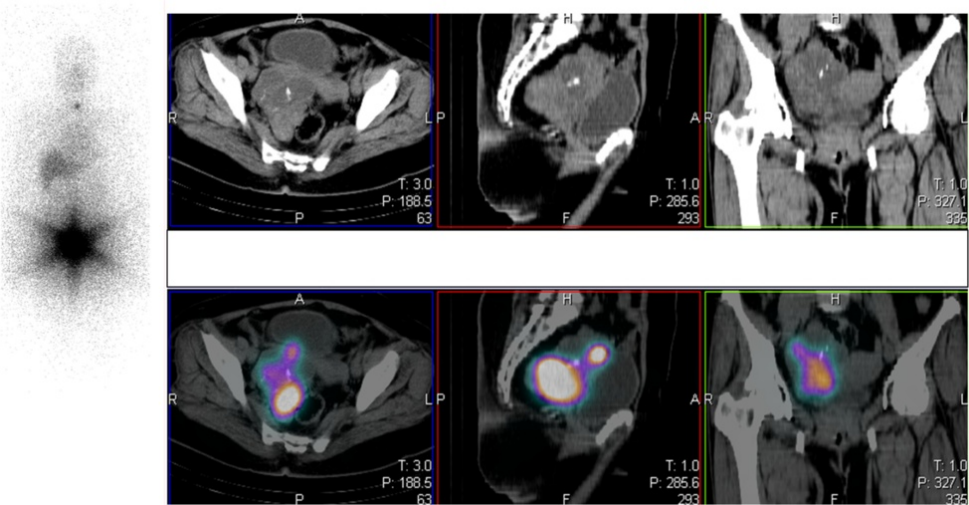
I^131^ whole-body scan after total thyroidectomy revealed a star artifact lesion in the pelvic area

Two weeks after total thyroidectomy, the patient’s symptoms of thyrotoxicosis recurred. The differential diagnosis included inadequate thyroidectomy or a source of extrathyroidal thyrotoxicosis such as functioning struma ovarii. A thyroid function test confirmed the presence of post-thyroidectomy thyrotoxicosis (Table 1). The radioactive iodine uptake was evaluated to check the adequacy of the thyroid surgery, and very low uptake of 0.2 % was found in the thyroid bed (reference range, 15 %–45 %). A radioactive iodine (I^131^) whole-body scan demonstrated intense radiotracer uptake with a star artifact in the pelvic region. Single-photon emission computed tomography/computed tomography of the pelvis confirmed the presence of inhomogeneous increased radiotracer uptake by an 8.5- × 7.2-cm mixed multicystic-solid mass with internal calcification in the right adnexal region (Fig. 2). The plasma level of cancer antigen 125 was elevated at 48.55 U/ml (reference range, 0–35 U/ml). Therefore the patient was diagnosed with coexisting Graves’ disease and functioning struma ovarii and surgery is scheduled. Preoperative control of thyrotoxicosis is required to prevent thyroid storm during the surgery. In the present case, therefore, the patient’s methimazole was restarted, and a euthyroid state was achieved before scheduling total abdominal hysterectomy with bilateral salpingo-oophorectomy (TAH with BSO).

**Table 1:** Thyroid hormone levels and management at baseline and during follow-up

	At first presentation	Before total thyroidectomy	2 weeks after total thyroidectomy	Before TAH with BSO	2 weeks after TAH with BSO
Free T_4_ (0.93–1.70 ng/dl)	–	1.14	4.8	1.43	0.11
Total T_4_ (4.50–11.70 μg/dl)	17.27	–	–	–	–
Total T_3_ (80–200 ng/dl)	233	167.5	380.7	153.2	–
TSH (0.27-4.20 mIU/ml)	<0.005	0.01	<0.005	0.16	>100
Treatment	Start propylthiouracil 300 mg/day	Methimazole 7.5 mg/day	Stop methimazole for 2 weeks	Methimazole 2.5 mg/day	Start levothyroxine 100 mcg/day

The patient’s perioperative course was uneventful. An 8.0- × 5.5- × 5.0-cm right ovarian mass with minimal ascites was found, the cut surfaces of the ovary showed solid-cystic appearance. The solid component showed soft red-brown and yellowish semitranslucent tissue resembling thyroid tissue. The cystic spaces contained clear yellow fluid.

**Fig. 3:**
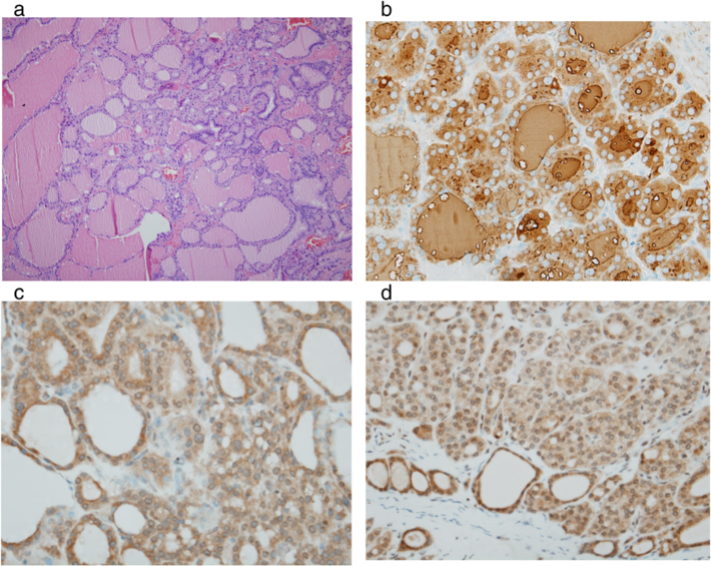
**a** H&E staining of our patient’s ovarian section revealed typical thyroid follicles. **b** The thyroid tissue in our patient’s ovarian tumor was highlighted by thyroglobulin antibody. **c** Immunostaining for TSH receptor showed positive staining in our patient’s struma ovarii. **d** TSH receptor also expressed on the tumor cells from other patient with non-functioning struma ovarii

The histological diagnosis was struma ovarii of the right ovary, without evidence of malignancy. Immunohistochemical staining for thyroglobulin (clone 2H11 + 6E1; Cell Marque) was positive; this result confirmed the thyroid epithelial nature of the lesion (Fig. 3b). Moreover, immunohistochemical staining for TSHR (clone 4C1/E1/G8; Abcam) was performed with normal thyroid tissue as a positive control and normal ovarian tissue as a negative control. Immunohistochemical staining was performed by autostainer (Ventana Benchmark XT). The result showed that the struma ovarii tissue in our patient was positive for TSHR, indicating the presence of TSHR expression in the struma ovarii tissue (Fig. 3c). Two weeks postoperatively, the patient’s thyroid hormone levels were in the hypothyroid range. Replacement therapy with levothyroxine was initiated, and euthyroidism was achieved.

## Discussion

Struma ovarii is a rare ovarian germ cell tumor which is entirely or predominantly composed of thyroid tissue [5]. Struma ovarii has been reported in 0.3 % to 1.0 % of all ovarian tumors and 2.0 % to 4.0 % of all ovarian teratomas [1]. It commonly occurs in the fourth to sixth decades of life, and it is usually benign and unilateral. Most affected patients are asymptomatic. However, patients may seek medical attention because of compressive symptoms involving a nearby organ, ascites, or even thyrotoxicosis. Thyrotoxicosis from struma ovarii has been reported in 5 % to 15 % of the confirmed cases [1–3, 6], but it is noted to be rare for tumors of <3 cm [7]. However, the actual incidence remains unclear owing to a lack of precise data on thyroid function in affected patients. Thyrotoxicosis in patients with struma ovarii has three potential causes: a hyperfunctioning struma ovarii alone, both a hyperfunctioning thyroid gland and a struma ovarii, or a hyperfunctioning thyroid gland with an incidental non-functioning struma ovarii [7]. The diagnosis of a functioning struma ovarii is challenging, especially in a patient with a hyperfunctioning thyroid gland. The evidence for the presence of a hyperfunctioning struma ovarii is based on increased radioiodine uptake by the ovary on an I^131^ whole-body scan [6]. Cervical goiter is quite common in patients with struma ovarii (16 %–40 %) [1, 2]. Because Graves’ disease is the most common cause of hyperthyroidism with diffuse thyroid gland enlargement, a number of previous cases of thyrotoxicosis due to struma ovarii were incorrectly treated by thyroidectomy [6, 7]. The coexistence of Graves’ disease and a hyperfunctioning struma ovarii is extremely rare. The characteristic of the prior case reports were summarized in Table 2. In most cases, the diagnosis was based on persistent postoperative thyrotoxicosis. In only two cases were both coexisting diseases simultaneously diagnosed [1, 8]. The optimal preoperative method for diagnosis of hyperfunctioning struma ovarii is either confirmation of the presence of thyrotoxicosis despite the absence of hyperfunctioning thyroid tissue or low radioiodine uptake at the neck along with high ovarian uptake of radioiodine tracer. Increased uptake of radioiodine by an ovarian tumor on a whole-body scan alone is insufficient for diagnosis of hyperfunctioning struma ovarii because it reportedly yields both false-positive and false-negative results [9–14]. The main differential diagnosis for increased uptake of radiotracer in the abdomen is ovarian metastasis from primary thyroid cancer.

**Table 2:** Cases of Coexisting Graves’ disease and Functioning Struma Ovarii

Author, year	Age at diagnosis	Presentation					TSH receptor antibody	Cervical thyroidectomy before diagnosis struma ovarii	Prior pelvic exam that result negative	Ovarian scan before abdominal surgery	Ovarian findings			Years after Graves’ disease diagnosis
		Exoph-thalmos	Thyroid bruit	Pelvic pressure symptoms	Pleural effusion	Ascites					Side	Maximum diameter (cm)	Malignancy	
Kampers, 1970 [7]	64	Yes	NA	NA	NA	NA	NA	Yes	NA	NA	NA	NA	No	18
Lefort, 1981 [9]	38	Yes	NA	NA	No	No	Positive	NA	NA	Positive	Right	7	No	12
Lazarus, 1987 [15]	48	Yes	NA	NA	NA	Few	Positive	Yes	14 years	Positive	Left	9	No	24
Kung, 1990 [10]	40	No	NA	No	NA	No	Positive	No	23 weeks	NA	Right	5	No	4
Banyot, 1995 [1]	30	No	No	Yes	NA	NA	Negative	No	NA	Positive	Right	7	NA	0
Grandet, 2000 [16]	78	NA	NA	NA	NA	Few	NA	Yes	NA	Positive	Bilateral	10	No	4
Kano, 2000 [8]	50	NA	NA	NA	NA	Yes	Positive	No	NA	NA	Left	7	Yes	0
Mimura, 2001 [6]	26	No	No	NA	NA	NA	Positive	No	NA	Positive	Left	16	No	4
Sussman, 2002 [17]	53	No	NA	No	NA	NA	NA	No	NA	NA	Right	7.5	Yes	5
Bartel, 2005 [18]	54	NA	NA	NA	NA	NA	Positive	Yes	NA	Positive	Left	NA	No	23
Guida, 2005 [11]	42	Yes	NA	NA	No	Marked	NA	Yes	NA	Negative	Right	12	No	1
Teale, 2006 [4]	36	Yes	NA	NA	NA	NA	Positive	Yes	NA	NA	Left	13.5	No	8
Chiofalo, 2007 [19]	42	No	NA	Yes	NA	Yes	Positive	Yes	NA	Positive	Right	12	No	0.5
Wong, 2009 [2]	44	Yes	Yes	Yes	NA	NA	Positive	Yes	NA	NA	Left	NA	Yes	1
Anastasilakis, 2013 [3]	49	No	NA	NA	Yes	Moderate	Positive	No	NA	NA	Right	18	No	2
Our case, 2015	56	Yes	No	No	No	Minimal	Positive	Yes	Not done	Positive	Right	8	No	23

Similar to most cases, the hyperthyroidism in our patient was thought to have arisen only from Graves’ disease based on typical signs of diffuse thyroid gland enlargement, bilateral exophthalmos, diffuse thyroid uptake with visualized activity in the pyramidal lobe, and an elevated level of plasma TSHR antibody. However, persistent post-thyroidectomy thyrotoxicosis with low I^131^ uptake at the thyroid bed and biochemical hypothyroidism after removal of the struma ovarii confirmed the diagnosis of a functioning struma ovarii.

The pathophysiology of thyrotoxicosis from struma ovarii remains unclear. The finding of tall thyroidal epithelium with scattered papillary infoldings, which is frequently found in struma ovarii, has a poor correlation with the presence of clinical thyrotoxicosis [7]. There are two proposed mechanisms underlying the pathophysiology of functioning struma ovarii. First, the ovarian tumor may have autonomous function, as in toxic multinodular goiter [7]. Second, in patients with coexisting Graves’ disease, TSHR antibodies stimulate the struma ovarii in the same way that they stimulate the thyroid tissue to cause Graves’ disease. Our patient’s ovarian section revealed typical thyroid follicles without features of malignancy. Immunohistochemical staining for the TSHR in the struma ovarii tissue was positive (Fig. 3c), thus supporting the second hypothesis [3, 4]. We performed TSHR staining in the struma ovarii of another patient with normal thyroid function test preoperatively and two other patients without confirm thyroid function test but was clinically euthyroid preoperatively. The staining results were all positive and the intensity of the TSHR staining of functioning strum ovarii was the same as that in other cases of nonfunctioning struma ovarii (Fig. 3d), suggesting that the determinant of functioning struma ovarii might be the presence of TSHR stimuli rather than the intensity of the TSHR in the ovarian tissue. In prior case reports of coexisting Graves’ disease and struma ovarii, the diagnosis of functioning struma ovarii almost always follows the diagnosis of Graves’ disease by several years. The circulating TSHR antibody in Graves’ disease presumably has a stimulatory effect on the thyroid tissue in the ovary, resulting in gradual growth and increased thyroid hormone production [3, 15].

Approximately 5 % of struma ovarii are malignant, regardless of whether they are functional, and tissue sampling for malignancy testing is very difficult. Therefore, struma ovarii should be surgically removed in all cases. Meanwhile, the coexisting Graves’ disease can be managed medically, surgically, or by radioactive iodine ablation.

## Conclusions

In patients with Graves’ disease with persistent or recurrent thyrotoxicosis after adequate ablative treatment, the possibility of ectopic thyroid hormone production such as that from struma ovarii or a metastatic differentiated thyroid cancer should be considered. TSHR expression is found in patients with functioning and non-functioning struma ovarii and cannot solely be used to determine the functioning status of the tumor.

## Consent

Written informed consent was obtained from the patient for publication of this case report and any accompanying images. A copy of the written consent is available for review by the editor of this journal.

## Abbreviations

TSHR: Thyroid-stimulating hormone receptor
I^131^: Radioactive iodine
TAH with BSO: Total abdominal hysterectomy with bilateral salpingo-oophorectomy
NA: Not available
